# Trypsin-, proteinase K-, and ribonuclease T1-functionalized magnetic nanoparticle-immobilized enzyme reactors based on polyelectrolyte multilayer platform for biopharmaceutical analysis

**DOI:** 10.1007/s00216-026-06580-5

**Published:** 2026-05-29

**Authors:** Yachao Hao, Niklas Carstensen, Ronny Loeffler, Benjamin Junker-Reiss, Dominique Lunter, Michael Lämmerhofer

**Affiliations:** 1 Institute of Pharmaceutical Sciences, Pharmaceutical (Bio-)Analysis, University of Tübingen, Auf Der Morgenstelle 8, 72076 Tübingen, Germany; 2 Center for Light-Matter Interaction, Sensors & Analytics (LISA+), University of Tübingen, Auf Der Morgenstelle 15, 72076 Tübingen, Germany; 3 Institute of Physical and Theoretical Chemistry, University of Tübingen, Auf Der Morgenstelle 15, 72076 Tübingen, Germany

**Keywords:** Magnetic nanoparticle, Sample preparation, Biopharmaceutical analytics, Immobilized enzyme reactor, Ribonuclease, Protease

## Abstract

Immobilized enzyme reactors (IMERs) have gained increasing attention owing to their numerous advantages, including reusability, easy handling, cost saving, and prolonged lifetime. In this work, Fe_3_O_4_ nanoparticles bearing different functional groups (-OH, -NH_2_, and -COOH) were modified with polyelectrolytes poly(ethyleneimine) and poly(acrylic acid), and systematically evaluated as magnetic supports for enzyme immobilization. Polyelectrolytes acting as spacer arms to minimize steric hindrance at the enzyme’s active sites and providing terminal carboxyl groups for covalent enzyme immobilization through amide coupling were selected for efficient surface chemistry. After optimization of the bonding chemistry, the synthesized IMERs were characterized using high-resolution surface imaging helium ion microscopy (HIM), Fourier transform infrared spectroscopy (FT-IR), zeta potential measurements, and energy dispersive X-ray spectroscopy (EDX). Trypsin, proteinase K, and ribonuclease (RNase) T1 as target enzymes were immobilized onto magnetic beads, respectively. The bioactivity of the immobilized enzymes was estimated by determination of the Michaelis-Menten parameters (*K*_*m*_, *V*_*max*_, *k*_*cat*_, and *k*_*cat*_*/K*_*m*_) of test substrates and compared to the corresponding enzyme in solution. The amount of bound enzyme was quantified by Micro BCA assay measuring the absorbance at 562 nm. Furthermore, the developed IMERs were applied to protein and RNA digestion, and the resulting fragments were analyzed by HPLC–ESI-QTOF-MS/MS, demonstrating their applicability for biomolecular analysis. Finally, successful mRNA digestion isolated from a vaccine documented the practical applicability of the RNase T1 IMER and the suitability of the polyelectrolyte multilayer enzyme immobilization platform.

**Graphical Abstract:**

**Figure:**
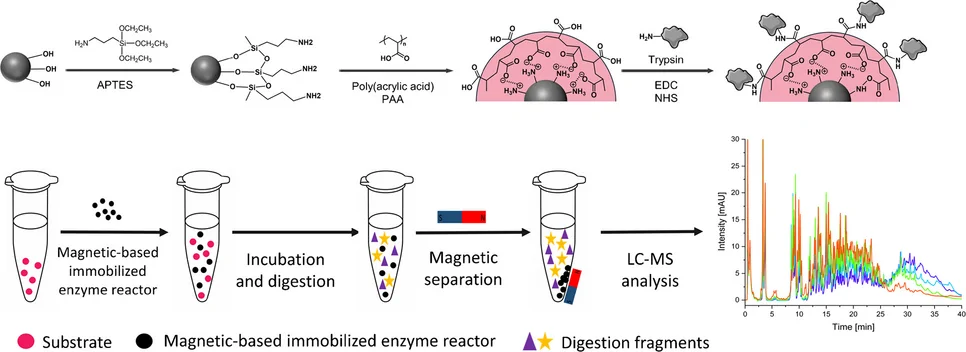

**Supplementary Information:**

The online version contains supplementary material available at https://doi.org/10.1007/s00216-026-06580-5.

## Introduction

Enzymes are biological catalysts that can catalyze a variety of biochemical reactions with high activity and specificity under mild conditions [1, 2]. For these reasons, they are also of great value for sample preparation in analytical chemistry. Enzymatic digestion is a fundamental process in biopharmaceutical analysis, where complex biomacromolecules were hydrolyzed into smaller, well-defined fragments that are better amenable to chromatographic separation and mass spectrometry (MS) characterization [3]. In proteomics, trypsin is the most commonly used protease due to its highly specific cleavage at the C-terminal end of lysine and arginine residues, generating tryptic peptides that can then be analyzed using LC-MS/MS analysis [4]. Likewise, in RNA analysis, enzymatic digestions are performed using ribonucleases such as RNase T1 to generate smaller oligoribonucleotides for RNA sequence mapping and modification analysis [5, 6]. However, the traditional enzymatic digestion is generally performed in solution, which is associated with many drawbacks such as enzyme autolysis, difficulty in recovery and reuse, low stability, and high sensitivity to employed conditions [7]. Moreover, after the digestion, the enzyme remains in the reaction mixture, leading to contamination of samples and analytical systems with protein, which could interfere with the subsequent analysis.

Immobilized enzyme reactors (IMERs) have been widely used as biocatalytic materials in bioanalysis via immobilizing enzymes onto solid supports as a powerful strategy to overcome the problems of in-solution digestion while maintaining their catalytic activity [7]. Various types of media, such as monolithic supports [8], lab-on-a-chip platforms [9], polymer films [10], open-tubular reactors [11], and micro- or nanobeads [12], have been utilized as IMER solid supports to avoid the detrimental interference from enzyme after the digestion reaction. Compared to free enzyme, immobilized enzymes exhibit higher stability and resistance to various environmental changes such as pH or temperature [12]. Furthermore, upon immobilization, the “site isolation” effect prevents the immobilized enzyme from digesting each other, which significantly reduces enzyme autolysis [13]. Recent advancements in nanotechnology have received considerable attention due to their great potential in biochemistry and materials science [14–16]. Nanoparticles are considered as an ideal support for enzyme immobilization because of their ultrahigh surface area, good mechanical stability, tunable surface chemistry, and high enzyme loading capability [16]. Magnetic nanoparticles [17, 18], silica nanoparticles [19, 20] and gold nanoparticles [21, 22] are the most commonly used nanoparticles for enzyme immobilization, via physical adsorption, cross-linking, covalent attachment, affinity binding, or encapsulation.

Among the nanoparticles, magnetic nanoparticles (MNPs) stand out due to their unique properties, including superparamagnetism, large surface-to-volume ratio, ease of surface modification, and rapid magnetic separation [23]. Magnetite (Fe_3_O_4_), in particular, is most commonly used as the core for MNP preparation and has been widely applied in many fields such as biosensing [24], drug delivery [25], tissue engineering [26], enrichment [27], environmental remediation [28] and enzyme immobilization [17]. The advantages of Fe_3_O_4_ include its ease of modification with functional groups such as hydroxyl (-OH), amino (-NH₂), carboxyl (-COOH), and other inorganic compounds, as well as its high affinity for proteins and good biocompatibility, making it a promising magnetic carrier for enzyme immobilization [18]. Furthermore, magnetite-based IMER technology allows for the simple and rapid separation of enzyme from the reaction medium by applying an external magnetic field, without being subjected to heavy mechanical stress compared with filtration or centrifugation [29]. Moreover, the reaction can be easily terminated via magnetic separation without the addition of stopping reagents, thus enabling instant reaction quenching and preventing overdigestion. Moreover, the resulting heterogeneous biocatalysts are easily recoverable and reusable, which is environmentally sustainable and economically interesting [30].

On the one hand, however, bare Fe_3_O_4_ nanoparticles are unstable and tend to spontaneously agglomerate to minimize surface energy because of magnetic dipole-dipole interactions [29], resulting in reduced dispersion and surface accessibility of MNPs as absorbents. On the other hand, straightforward enzyme immobilization onto unmodified Fe_3_O_4_ surfaces can cause the active sites of enzyme to be partially blocked or sterically hindered by the magnetic nanoparticles and adjacent enzyme molecules, thus reducing the bioactivity of immobilized enzyme [30, 31]. To address both aggregation and activity loss, polyelectrolytes present outstanding advantages as spacer arm and play crucial roles in surface modification and enzyme immobilization [32–34]. Such charged polymeric layers can not only prevent magnetic nanoparticles aggregation through electrostatic repulsion and steric hindrance, but also improve flexibility of immobilized enzyme and reduce steric constraints around their active sites, thereby increasing catalytic efficiency [33].

Thus, in this work, Fe_3_O_4_ nanoparticles bearing different functional groups were modified with polyelectrolytes, which act as a spacer arm to improve stability and enzyme accessibility to provide a platform for immobilization of various enzymes. The polyelectrolytes also provided active carboxylic groups as the terminal moiety, which can be covalently coupled with the amino group of the enzyme to avoid enzyme leaching from the magnetic support. The bioactivity of immobilized enzyme and the loading amount of protein of different IMERs, as well as the batch-to-batch reproducibility, reusability, and storage stability of the immobilized trypsin systems, were systematically investigated. In peptidomics analysis, peptides are cleaved by a series of unknown endogenous proteolytic enzymes, which requires searching against all possible peptide fragments [35–37]. Therefore, in this study, proteinase K as the non-specific protease was further immobilized onto the optimized IMER to assess its potential for generating diverse short peptide mixtures applicable to peptidomics profiling. In addition, RNase T1 was immobilized to expand the application of the developed IMER system. Finally, the optimized magnetic IMER combined with HPLC–ESI-QTOF-MS/MS method was successfully applied for biomolecular analysis. In this study, we present a comprehensive characterization and functional evaluation of a novel polyelectrolyte-based magnetic IMER platform across diverse enzyme classes and for both protein and RNA digestion, followed by LC-MS analysis. Unlike conventional approaches that focus on single enzyme immobilization, our work offers new insights by demonstrating the immobilization of multiple enzymes on the same platform and their application across distinct molecular domains, i.e., proteins and nucleic acids.

## Materials and methods

### Chemicals and reagents

Trypsin (from porcine pancreas, E.C.3.4.21.4, 1670 U mg^−1^) and Cytochrome c (CYC_HORSE, from horse heart, MW 12,384 Da) were obtained from Sigma-Aldrich (Munich, Germany). Proteinase K (from Tritirachium album, E.C.3.4.21.64, ≥ 30U mg^−1^) was obtained from Carl Roth (Karlsruhe, Germany). RNase T1 endoribonuclease (E.C.3.1.27.3, 1000 U μL^−1^) and Micro BCA assay kit for determination of the amount of immobilized enzyme were purchased from Thermo-Fisher Scientific (Karlsruhe, Germany). RNA oligonucleotide test sample (AUCGGCCAUGCGAAU) was synthesized by Oligo Sigma (Merck, Munich, Germany) and purchased as desalted raw-products. mRNA vaccine Comirnaty Omicron XBB.1.5 (raxtozinameran) was purchased from Pfizer (New York, USA).

FeCl_3_⋅6H_2_O, FeCl_2_⋅4H_2_O, ammonia, 3-aminopropyltriethoxysilane (APTES), glutaric anhydride, *N*,*N*-dimethylformamide (DMF), poly(acrylic acid, sodium salt) solution (PAA, Mw. ~ 15,000, 35wt. % in H_2_O), poly(ethyleneimine) solution (PEI, Mw. ~ 750,000, 50wt. % in H_2_O), *N*-(3-dimethylaminopropyl)-*N*′-ethylcarbodiimide (EDC), *N*-hydroxysuccinimide (NHS), *N*-benzoyl-DL-arginine 4-nitroanilide hydrochloride (BApNA), hydrochloric acid (37%) (HCl), Tween 20, glycerol, ethanol, isopropanol, phosphate-buffered saline (PBS), tris(hydroxymethyl)aminomethane (Tris), ammonium bicarbonate, and 2-(*N*-morpholino) ethanesulfonic acid (MES) monohydrate were all obtained from Sigma-Aldrich (Munich, Germany). Sera-Mag carboxylate-modified magnetic beads as a benchmark was obtained from Cytiva (Freiburg, Germany). Acetonitrile, formic acid (FA), trifluoroacetic acid (TFA), and ammonium acetate from Carl ROTH (Karlsruhe, Germany) were of LC-MS grade. Deionized water (produced by Elga Purelab Ultra ELGA, System LabWater, Celle, Germany) was used throughout the study.

### Instruments

Liquid chromatographic separation was performed with an Agilent 1290 HPLC system equipped with a binary gradient pump, a vacuum degasser, a thermostated column oven, and a UV detector (Agilent, Waldbronn, Germany). Sample injection was performed using a CTC-PAL HTS autosampler (CTC Analytics, Zwingen, Switzerland). Mass spectrometric detection was carried out on a TripleTOF 5600+ quadrupole time-of-flight mass spectrometer (Sciex, Concord, Canada) fitted with a DuoSpray source operated in electrospray ionization (ESI) mode.

The particle size and morphology of the materials were characterized using the Helium Ion Microscope ORION NanoFab (Carl Zeiss, Oberkochen, Germany) operated with an acceleration voltage of 30 kV and a beam current of 0.35 pA. The system enabled sub-nanometer resolution imaging with high surface sensitivity. Fourier transform infrared spectroscopy was performed on an evacuated Bruker Vertex 70v FT-IR spectrometer (Bruker, Germany) using the KBr press-pellet method. The zeta potential of the materials was determined by electrophoretic light scattering measurements using a Zetasizer Nano ZS (Malvern Instruments, Herrenberg, Germany) equipped with folded capillary cells and all experiments were performed in triplicates. Trypsin activity was measured with a VersaMax microplate reader (Molecular Devices, Biberach, Germany).

### Synthesis of hydroxyl-, amino-, and carboxyl-functionalized Fe_3_O_4_

The Fe_3_O_4_ was prepared via a coprecipitation method following a similar procedure to that reported by Ref. [38]. 5.4 g of FeCl_3_⋅6 H_2_O and 2 g of FeCl_2_⋅4H_2_O were dissolved in a molar ratio of 2:1 in 100 mL deionized water and stirred for 30 min at 80 °C under nitrogen atmosphere. Then, ammonia solution was added dropwise to adjust the pH to 10 and the mixture stirred for an extra 30 min. Afterwards, the product was separated with a magnet and washed separately with deionized water and ethanol. After vacuum drying at 60 °C for 8 h, black Fe_3_O_4_-OH nanoparticles were obtained by grinding. For the synthesis of Fe_3_O_4_-NH_2_, 1 g of Fe_3_O_4_ was dissolved in 100 mL of ethanol and sonicated for 30 min. Then, 1 mL of APTES was added to the system using a pipette, and heated to 50 °C with mechanical stirring overnight [39]. The carboxyl-functionalized Fe_3_O_4_ nanoparticles (Fe_3_O_4_-COOH) were synthesized via surface functionalization of Fe_3_O_4_ nanoparticles with APTES and glutaric anhydride according to a reported method [40].

### Polyelectrolyte modification onto Fe_3_O_4_-OH, Fe_3_O_4_-NH_2_, and Fe_3_O_4_-COOH

The polyelectrolyte as the spacer arm was coated onto magnetic support to introduce terminal carboxyl groups for enzyme immobilization [22]. For Fe_3_O_4_-NH_2_, 50 mg of corresponding MNPs were dispersed in 10 mL deionized water (pH adjusted to 6 with 1 M HCl) and ultrasonicated, then 10 mL of PAA solution (20 mg mL^−1^) was added and stirred vigorously for 45 min at room temperature. Afterwards the supernatant was discarded, the pellet was collected by a magnet and washed twice with deionized water. The modified magnetic beads (Fe_3_O_4_-NH_2_/PAA) were resuspended in 10 mL of 20 mM phosphate buffer (pH 6.8) and stored at 4 °C for further usage. For Fe_3_O_4_-OH and Fe_3_O_4_-COOH, 50 mg of corresponding MNPs were dispersed in 10 mL deionized water and ultrasonicated, then 10 mL of PEI solution (20 mg mL^−1^) as the first layer and 10 mL of PAA solution (20 mg mL^−1^) as the second layer were coated onto Fe_3_O_4_-COOH according to the above steps. Finally, Fe_3_O_4_-OH/PEI/PAA and Fe_3_O_4_-COOH/PEI/PAA were stored in 10 mL of 20 mM phosphate buffer (pH 6.8) at 4 °C for further usage.

### Immobilization of Trypsin, Proteinase K and RNase T1 onto magnetic supports

**Fig.1:**
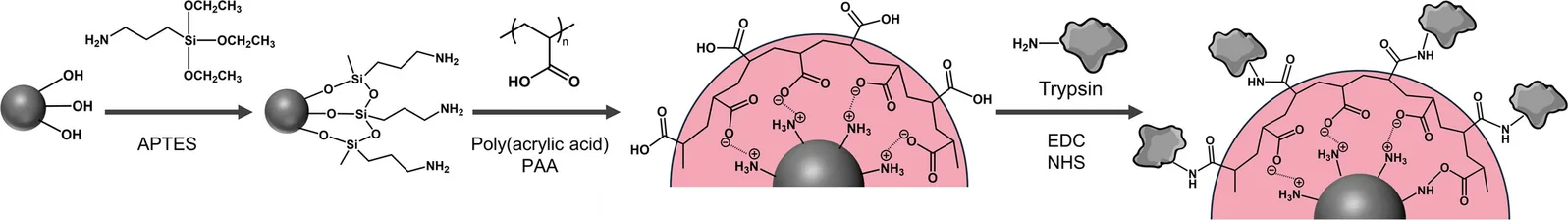
Schematic diagram of the preparation of Fe_3_O_4_-NH_2_/PAA/trypsin

Fe_3_O_4_-OH/PEI/PAA, Fe_3_O_4_-COOH/PEI/PAA, Fe_3_O_4_-NH_2_/PAA, and commercial carboxylate-modified magnetic beads (Cytiva, as a benchmark) were comparatively assessed for enzyme immobilization, and the support showing the highest retained activity was chosen for further studies. Briefly, magnetic beads were washed and dispersed in 500 μL deionized water. Then, 250 μL of 200 mM MES buffer (pH 4.5) was added, followed by activation of surface carboxyl groups with 250 μL of EDC (40 mg mL^−1^) and 250 μL of NHS (10 mg mL^−1^) [41]. The mixture was stirred at room temperature for 30 min to generate the active ester intermediates. Activated particles were magnetically separated to remove excess reagents. Subsequently, the activated magnetic beads were resuspended in 20 mM PBS buffer (pH 7.4), enzyme (concentrations in the reaction mixture see below) was added and stirred for 30 min at room temperature for immobilization. After immobilization, the supernatant was discarded and the obtained IMER was washed with 20 mM PBS buffer (pH 7.4) containing 0.1% (w/v) Tween-20 to remove non-specifically absorbed enzyme. The washing procedure was repeated six times and the IMER solution was stored at 4 °C prior to use. This way, immobilized trypsin (500 μg mL^−1^), immobilized proteinase K (200 μg mL^−1^), and immobilized RNase T1 (50 U μL^−1^) were prepared, respectively. The schematic preparation process of IMER using Fe_3_O_4_-NH_2_/PAA/trypsin as the example is shown in Fig. 1.

### Enzyme activity assays and kinetic parameters

#### Assay of trypsin activity

The chromogenic standard BApNA was selected as substrate to determine the activity of free trypsin and immobilized trypsin. BApNA was cleaved by trypsin to produce p-nitroaniline (pNA) which exhibits an absorption at 405 nm [18]. A concentration series of BApNA (10, 5, 2, 1, and 0.5 mM) was digested with trypsin in 50 mM Tris-HCl buffer (pH 8.0) at 37 °C. For each assay, 100 μL of substrate solution was mixed with 100 μL of 500 μg mL^−1^ free trypsin or immobilized trypsin (250 μg mL^−1^ final concentration). After 10 min incubation, the immobilized trypsin was separated by a magnet and then 150 µL of supernatant was pipetted into 96-well plate and analysed using a Versa max microplate reader in endpoint mode at 405 nm. All experiments were performed in triplicate. The Michaelis-Menten parameters *K*_*m*_, *V*_*max*_, and *k*_*cat*_ were calculated by Lineweaver-Burk equation. The catalytic activities of the in-house synthesized immobilized trypsin were evaluated against those of free trypsin and benchmark carboxy-terminated magnetic beads (trypsin immobilization performed by the same procedure through EDC/NHS amide coupling described above), and the support providing the highest activity was selected for subsequent applications.

#### Assay of proteinase K activity

Cytochrome c as the model substrate was selected to determine the activity of free proteinase K and immobilized proteinase K at 50 °C in 50 mM Tris-HCl buffer (pH 8.0). Enzymatic reaction was performed using different concentrations of CYC and 200 µg mL^−1^ proteinase K solution (50 µg mL^−1^ final concentration). After 10 min of digestion, formic acid (final concentration 1%) was added to terminate the reaction catalyzed by free proteinase K, and the reaction with immobilized proteinase K was stopped by magnetic separation. The supernatant was then subjected to LC-UV analysis at 214 nm for the quantification of undigested CYC. Then the kinetic parameters were calculated according to Lineweaver-Burk plot.

### Determination of the amount of immobilized protein

The amount of immobilized protein was determined using the Micro BCA Protein Assay Kit following the manufacturer’s instructions. The working reagent was freshly prepared by mixing Micro BCA reagent A, B, and C with a ratio of 25:24:1. A calibration curve was established using bovine serum albumin (BSA) standard included in the Kit, within the concentration range of 0.5–20 µg mL^−1^, corresponding to the linear working range of Micro BCA assay kit (Fig. S4). Briefly, a 20 µL aliquot of the blank/sample/standard was diluted with 480 µL of deionized water and then mixed with 500 µL of working reagent. After incubation at 60 °C for 60 min, the immobilized enzyme was magnetically separated, and the absorbance of the supernatant was measured at 562 nm. The absorbance signal was correlated to the amount of immobilized protein through the calibration function.

The characterization results of the amount of immobilized protein on different IMERs are summarized in Tables S1–S4. And a more detailed discussion regarding the adsorption mechanism can be found in the Electronic Supplementary Material.

### Protein digestion

Cytochrome c (CYC) was utilized as a model protein to evaluate the digestion efficiency of immobilized trypsin. The immobilized trypsin was first washed and resuspended in 50 mM ammonium bicarbonate buffer (pH 8.0) to ensure MS compatibility. For digestion, 50 µL of Fe_3_O_4_-NH_2_/PAA/trypsin suspension was mixed with 100 µL of CYC solution (1 mg mL^−1^), followed by 50 µL of digestion buffer, and incubated at 37 °C on a Thermoshaker at 1100 rpm (Peqlab, Erlangen, Germany) for 10 min, 30 min, 1 h, 4 h, and 16 h. For comparison, free trypsin was employed under the same conditions. After digestion, 2 µL of formic acid was added to terminate the reaction catalyzed by free trypsin, and the reaction with immobilized trypsin was stopped by magnetic separation. The resulting supernatants were collected for HPLC-ESI-QTOF-MS analysis.

### RNA digestion

A 15-mer RNA oligonucleotide (AUCGGCCAUGCGAAU) ordered from Merck was employed as a model substrate to assess the digestion efficiency of immobilized RNase T1. For the digestion assay, 20 µL of RNA standard (20 µM) was mixed with 20 µL of immobilized RNase T1 solution (50 U μL^−1^), followed by the addition of 60 µL digestion buffer to obtain a final reaction volume of 100 µL. After the reaction, the immobilized RNase T1 was magnetically separated, and the resulting supernatant was collected for LC-ESI-QTOF-MS analysis to identify the digestion fragments of RNA standard.

### HPLC-ESI-QTOF-MS method

Trypsin and proteinase K digested samples were separated by an Agilent 1290 HPLC system (Waldbronn, Germany) using an Aeris Peptide C18 column (150 × 2.1 mm, 3.6 μm) from Phenomenex (Torrance, CA, USA). The mobile phase consisted of H_2_O with 0.1% (v/v) formic acid in channel A and acetonitrile with 0.1% (v/v) formic acid in channel B (0–2 min, 5% B; 2–12 min, 5–60% B; 12–12.1 min, 60–5% B; 12.1–15 min, 5% B). The flow rate was 0.4 mL min^−1^, injection volume was 5 μL, and column temperature was set to 40 °C. The peptide masses were detected with a Triple TOF 5600+ mass spectrometer from Sciex (Ontario, Canada) in ESI positive mode. Data were acquired with a TOF-MS full scan combined with information-dependent acquisition (IDA) in high-sensitivity mode. TOF-MS full scan was acquired with an accumulation time of 250 ms and a scan range from 100 to 2000 *m/z*. IDA-MS/MS experiments were performed with an accumulation time of 25 ms, precursor ions with *m/z* > 100 and an intensity threshold greater than 100 counts per second were selected for MS/MS acquisition. A maximum of 10 candidate precursor ions were selected per cycle. MS parameters were set as follows: curtain gas: 30 psi; nebulizing gas (GS1): 50 psi; drying gas (GS2): 40 psi; ion spray voltage floating (ISVF): 5500 V; source temperature (TEM): 450 °C; declustering potential: 100 V. The dynamic background subtraction and rolling collision energy were activated.

For data analysis, Mascot MS/MS ions search was performed for protein identification against SwissProt database. Trypsin was specified as the digestion enzyme, allowing a maximum of one missed cleavage. The peptide mass tolerance was set to ± 0.5 Da and the MS/MS fragment ion tolerance to ± 0.6 Da. Raw data files were converted into Mascot generic format (.mgf) using MSConvertGUI prior to database searching.

The RNase T1 digested RNA standard were separated by an Acquity UPLC BEH Amide (150 × 2.1 mm, 1.7 μm) from Waters (Eschborn, Germany). The mobile phase consisted of H_2_O with 10 mM ammonium acetate at pH 8.2 in channel A and 90/10 (v/v) acetonitrile/water with 10 mM ammonium acetate in channel B. The gradient profile was as follows: 0–2 min, 10% A at 0.3 mL min^−1^; 2–15 min, 10–55% A at 0.3 mL min^−1^; 15–16 min, 55% A at 0.3 mL min^−1^; 16–16.1 min, 10% A at 0.5 mL min^−1^; 16.1–22 min, 10% A at 0.5 mL min^−1^. Mass spectrometric detection was performed in negative polarity mode with an ESI source. Data were recorded by information-dependent acquisition (IDA): TOF-MS full scan with an accumulation time of 250 ms, scan range from *m/z* 100–3000, followed by IDA-based MS/MS acquisition of up to 5 precursor ions per cycle with an accumulation time of 25 ms per MS/MS scan. MS parameters were set as follows: curtain gas: 30 psi; nebulizing gas (GS1): 50 psi; drying gas (GS2): 40 psi; ion spray voltage floating (ISVF): −4500 V; source temperature (TEM): 550 °C; declustering potential: −250 V. Data processing and identification of RNA digests were performed by using PeakView (AB Sciex, Darmstadt, Germany).

### mRNA extraction and digestion

Due to the low concentration of mRNA in Comirnaty (100 µg/mL), mRNA was extracted and concentrated from the mRNA-LNP formulation to obtain high-quality purified sample for subsequent analysis. The extraction was performed by isopropanol precipitation according to the United States Pharmacopeia (USP) guideline with slight modifications [42]. Briefly, 200 µL of the vaccine solution was precipitated with 1800 µL of pure isopropanol containing 60 mM ammonium acetate. The samples were then centrifuged at 15,000 × g for 15 min at 4 °C. After removal of supernatant, the pellet was washed with 1 mL of isopropanol and 70% ethanol successively, dried in a vacuum concentrator for 20 min at 30 °C and then resuspended in 20 µL of nuclease-free water resulting in a nominal tenfold concentration of the sample.

For the digestion assay, 20 µL of the extracted mRNA was mixed with 20 µL of immobilized RNase T1 solution (100 U μL^−1^), followed by the addition of 10 µL digestion buffer to obtain a final reaction volume of 50 µL. The digestion was carried out at different digestion times on a thermoshaker at 1500 rpm and 37 °C. After digestion, the reaction was terminated by magnetic separation. The digested mRNA was diluted 1:1 with acetonitrile and then injected for HILIC-UV analysis.

## Result and discussion

### Characterization of materials

**Fig. 2:**
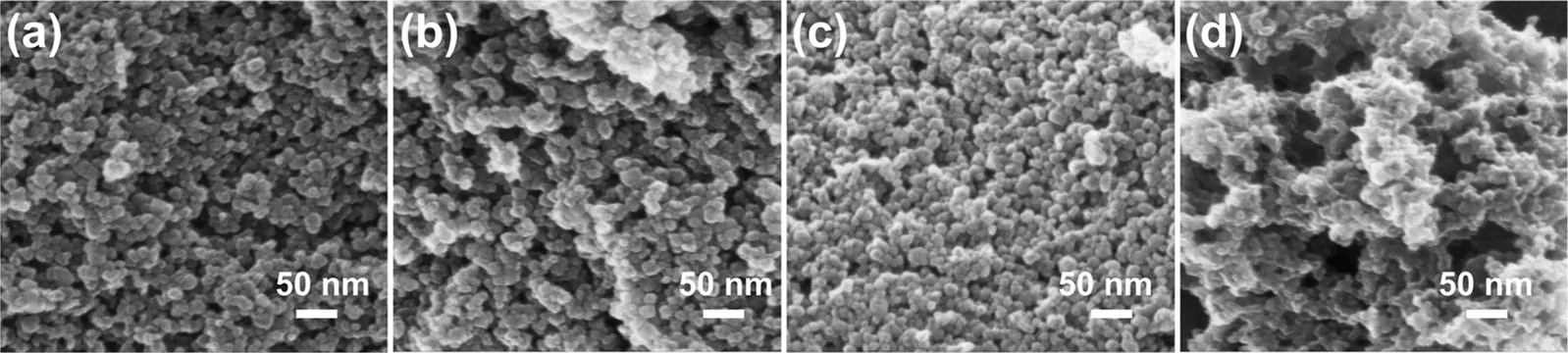
HIM images of Fe_3_O_4_ (**a**), Fe_3_O_4_-NH_2_ (**b**), Fe_3_O_4_-NH_2_/PAA (**c**), and benchmark (**d**)

The morphology and particle size of the synthesized magnetic nanoparticles were characterized by HIM. As shown in Fig. 2a and b, the bare Fe_3_O_4_ nanoparticles with -OH groups and the APTES-modified Fe_3_O_4_ nanoparticles with -NH_2_ groups exhibited near-spherical but slightly irregular particle shape. Besides, the images showed the formation of some agglomerates probably due to the magnetic dipole moment interaction between particles [29]. The mean diameter of the Fe_3_O_4_ nanoparticles was approximately 15 nm. Following surface modification of Fe_3_O_4_-NH_2_ with PAA (Fig. 2c), it can be seen that the nanoparticles exhibited a well-shaped spherical morphology and particle distribution became more uniform. Particle size slightly increased. This result confirms the favorable effect of the polymeric coating in improving the stability and dispersion of Fe_3_O_4_ nanoparticles. Figure 2d shows the HIM image of commercially available carboxylate-modified magnetic beads from Cytiva, which were used as a comparative benchmark in this study. Although the specific modification is not disclosed, it appears to have a smaller average particle size than the in-house synthesized magnetic beads and have a superficially porous surface structure. HIM results illustrated that the magnetic support Fe_3_O_4_-NH_2_/PAA for enzyme immobilization was prepared successfully.

**Fig. 3:**
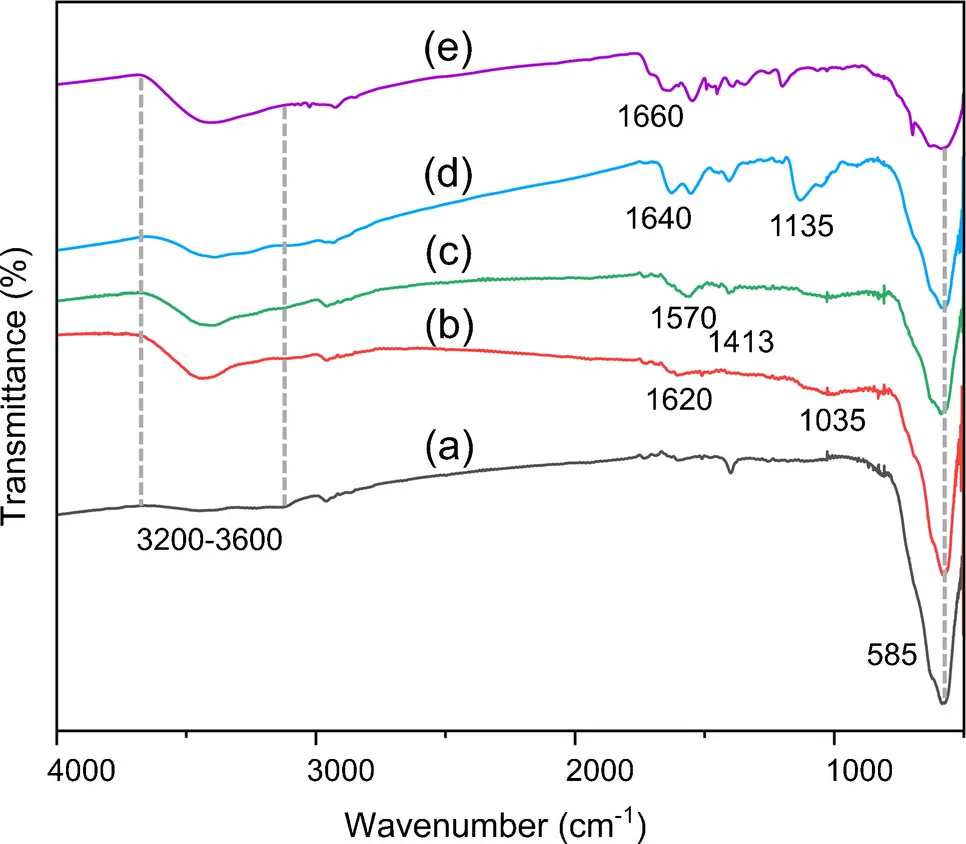
FT-IR images of Fe_3_O_4_ (**a**), Fe_3_O_4_-NH_2_ (**b**), Fe_3_O_4_-NH_2_/PAA (**c**), Fe_3_O_4_-COOH (**d**), and benchmark (**e**)

The FT-IR analysis (Fig. 3) was performed to analyse the surface functionalization of Fe_3_O_4_ MNPs before and after the chemical modification. The absorption peak at 585 cm^−1^ observed in all samples was due to the characteristic Fe-O stretching vibrations, which confirmed the presence of magnetite nanoparticles [43]. In Fig. 3a, the broad bands between 3600 and 3200 cm^−1^ indicated the presence of hydrogen-bonded hydroxyl groups (O-H stretching vibrations) at the Fe_3_O_4_ surface. For Fe_3_O_4_-NH_2_, an enhanced band in the frequency range of 3400–3300 cm^−1^ and the peak at 1620 cm^−1^ were attributed to the N-H stretching and bending vibrations, respectively. The broad peak around 1035 cm^−1^ corresponds to Si-O stretching vibration, indicating the coupling agent APTES had been introduced onto the Fe_3_O_4_ surface. After PAA modification (Fig. 3c), the absorption peaks at 1570 cm^−1^ and 1413 cm^−1^ were attributed to the asymmetric and symmetric stretching vibrations of carboxylate groups (-COO^−^), respectively [43, 44]. This indicates PAA was modified through electrostatic interaction between -COO^−^ of PAA and -NH_3_^+^ groups on Fe_3_O_4_ surface rather than existing as free carboxylate pairs. Moreover, the intensity of absorption peak at 585 cm^−1^ was lower than that of Fe_3_O_4_-NH_2_, which further confirmed the successful modification of PAA on the surface of Fe_3_O_4_-NH_2_. For Fe_3_O_4_-COOH (Fig. 3d), the absorption peak at 1640 cm^−1^ corresponds to C = O stretching vibration of -COOH group (shifted to lower wavenumbers when coordinated to the Fe_3_O_4_ surface), and the peak at 1135 cm^−1^ was attributed to C-O stretching vibration, confirming the presence of carboxylic acid groups at the surface of Fe_3_O_4_. In Fig. 3e, the characteristic absorption peak of C = O stretching vibration was observed at 1660 cm^−1^, which indicated the formation of carboxylate modified magnetic beads used as benchmark material. In addition, the FT-IR spectra of the other magnetic supports, Fe_3_O_4_-OH/PEI/PAA and Fe_3_O_4_-COOH/PEI/PAA, are presented in Fig. S1. In Fig. S1(b) and (e), the broad peak between 3500 and 3300 cm^−1^ and the peak around 1008 cm^−1^ correspond to N-H stretching vibrations and C-N stretching vibration, confirming the successful modification of PEI layer. After the second-layer PAA modification, the characteristic bands at 1554 cm^−1^ and 1403 cm^−1^ were attributed to the asymmetric and symmetric stretching vibrations of -COO^−^ (Fig. S1(c)), indicating the successful synthesis of Fe_3_O_4_-OH/PEI/PAA. For Fe_3_O_4_-COOH/PEI/PAA, in Fig. S1(f), the asymmetric stretching vibration of -COO^−^ at 1560 cm^−1^ and the enhanced broad O-H absorption band in the range of 3200–3600 cm^−1^ indicate the introduction of additional carboxyl groups from PAA. The characteristic -COO^−^ bands do not show significant changes after PAA deposition, which may be due to the overlap with pre-existing carboxyl-related bands of Fe_3_O_4_-COOH as well as the formation of polyelectrolyte multilayer [45].

The surface charge of magnetic nanoparticles after each step of synthesis and surface modification was characterized by zeta potential measurements. As shown in Fig. S2, the initial zeta potential of Fe_3_O_4_-OH was 39.9 mV. After PEI modification, the zeta potential further increased to 44.5 mV, as expected for successful PEI coating. Following deposition of the oppositely charged PAA as the second layer, the zeta potential reversed to a negative value (−46.5 mV), indicating successful functionalization of negatively charged carboxyl groups via electrostatic interaction. Fe_3_O_4_-NH_2_ exhibited a positive surface potential value of 38.3 mV due to protonated amine groups, which reversed to −43.5 mV after PAA modification, confirming the successful synthesis of Fe_3_O_4_-NH_2_/PAA. Similarly, Fe_3_O_4_-COOH initially presented a negative zeta potential (−26.5 mV), after sequential modification with PEI and PAA, the zeta potential changed from the positive value of 37.4 mV to the negative value of −42.8 mV, demonstrating the successful layer-by-layer assembly of PEI and PAA. It is worth noting that although Fe_3_O_4_-OH was positively charged under the experimental conditions, PEI modification can still occur. This is likely driven by strong hydrogen bonding between the abundant amine groups of PEI and surface hydroxyl groups, as well as coordination interactions with surface Fe sites, enabling effective surface functionalization. Moreover, the increased absolute zeta potential values after polyelectrolyte modification indicate improved dispersion stability of the magnetic supports.

EDX analysis was performed to provide qualitative determinations of the elemental composition. The EDX spectra of Fe_3_O_4_-NH_2_/PAA/trypsin, Fe_3_O_4_-NH_2_/PAA/proteinase K, and Fe_3_O_4_-NH_2_/PAA/RNase T1 confirmed the presence of Fe, Si, N, C, and O (Fig. S3).

### Application I: Trypsin-IMER for protein digestion

#### Kinetic study and comparison of different IMERs

**Fig. 4:**
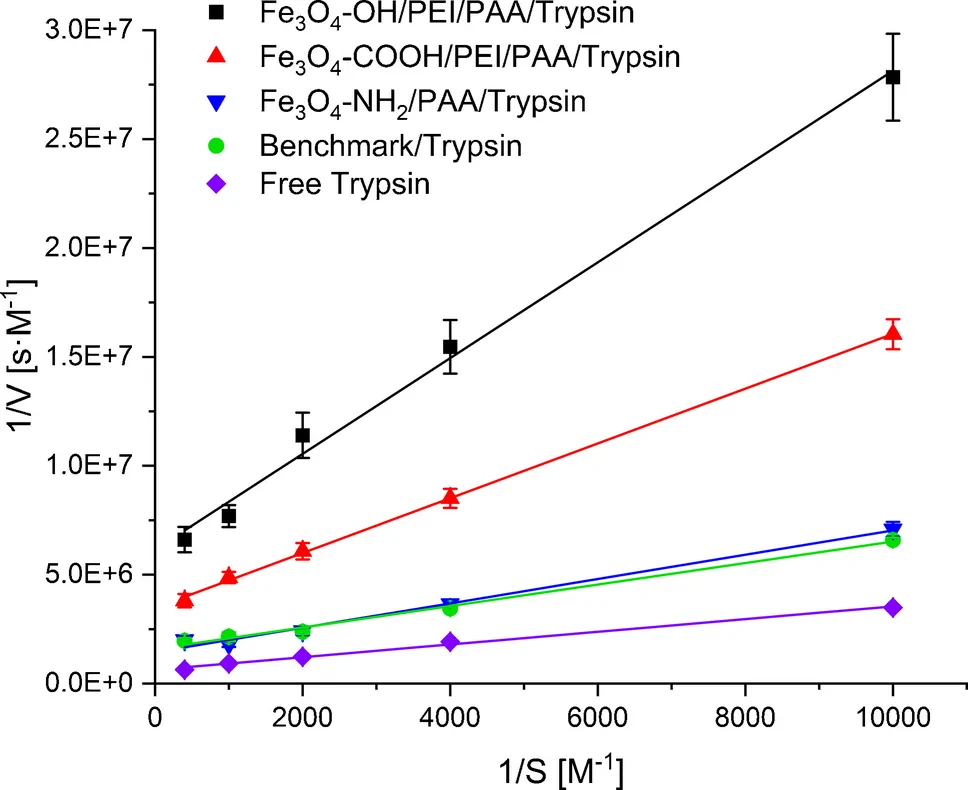
Lineweaver-Burk diagrams of different IMER-trypsin and free trypsin

Trypsin was immobilized onto Fe_3_O_4_-OH/PEI/PAA, Fe_3_O_4_-NH_2_/PAA, Fe_3_O_4_-COOH/PEI/PAA and commercial carboxylate-modified magnetic beads as a benchmark. The catalytic performance of the immobilized trypsin and free trypsin was evaluated using BApNA as the substrate by measuring the absorbance at 405 nm. The corresponding Michaelis-Menten parameters (*K*_*m*_, *V*_*max*_, *k*_*cat*_, and *k*_*cat*_*/K*_*m*_) were determined and the results are shown in Table 1 and Fig. 4.

**Table 1:** Comparison of kinetic parameters for different trypsin-IMER and free trypsin

Parameter	Fe_3_O_4_-OH/PEI/PAA/trypsin	Fe_3_O_4_-COOH/PEI/PAA/trypsin	Fe_3_O_4_-NH_2_/PAA/trypsin	Benchmark/trypsin	Free Trypsin
*K*_*m*_ [M]	(3.58 ± 0.20) × 10^–4^	(3.61 ± 0.32) × 10^–4^	(3.87 ± 0.54) × 10^–4^	(3.10 ± 0.30) × 10^–4^	(4.64 ± 0.41) × 10^–4^
*V*_*max*_ [M·s^−1^]	(1.63 ± 0.06) × 10^–7^	(2.87 ± 0.19) × 10^–7^	(6.93 ± 0.77) × 10^–7^	(6.29 ± 0.52) × 10^–7^	(1.59 ± 0.12) × 10^–6^
*K*_*cat*_ [s^−1^]	(6.89 ± 0.04) × 10^–2^	(1.32 ± 0.10) × 10^–1^	(2.49 ± 0.31) × 10^–1^	(2.77 ± 0.24) × 10^–1^	(1.51 ± 0.11) × 10^–1^
*K*_*cat*_*/K*_*m*_ [M^−1^·s^−1^]	(1.93 ± 0.15) × 10^2^	(3.66 ± 0.43) × 10^1^	(6.44 ± 1.20) × 10^2^	(8.94 ± 1.20) × 10^2^	(3.26 ± 0.38) × 10^2^
Final trypsin concentration [M]	(2.36 ± 0.09) × 10^–6^	(2.17 ± 0.09) × 10^–6^	(2.78 ± 0.15) × 10^–6^	(2.27 ± 0.07) × 10^–6^	1.05 × 10^–5^

*K*_*m*_ and *V*_*max*_ values were obtained via the Lineweaver-Burk fitting (Eq. (1)) and the turnover number (*k*_*cat*_) was calculated from Eq. (2):

1 $$\frac{1}{{V}_{0}}=\frac{{K}_{m}}{{V}_{max}[S]}+\frac{1}{{V}_{max}}$$

2 $${K}_{cat}=\frac{{V}_{max}}{[E]}$$

where V_0_ (M s^−1^) is the initial velocity of the enzyme, and [S] (mol L^−1^) and [E] (mol L^−1^) are the substrate and enzyme concentration, respectively.

*K*_*m*_ is the substrate concentration at which the reaction rate is half of *V*_*max*_, reflecting the affinity of enzyme for substrate. It is an intrinsic kinetic parameter that is independent of enzyme concentration. Upon immobilization, *K*_*m*_ might change as a result of the conformational change of the enzyme and the steric hindrance to enzyme active sites caused by support and adjacent enzymes, which may limit the access of the enzyme active sites for substrate [46]. It can be seen from Table 1 that *K*_*m*_ values of all immobilized trypsin remain in the same order of magnitude with free trypsin in solution (3.10–4.64 × 10^–4^ M), indicating that the polymer spacer layers provided sufficient flexibility to minimize steric hindrance at enzyme active sites and did not compromise substrate binding affinity.

The *V*_*max*_ is the maximal reaction rate achieved when enzyme is fully saturated with substrate and is proportional to the enzyme concentration. After trypsin was immobilized onto magnetic beads, the unbound and non-covalently bound trypsin was washed with detergent. Therefore, the *V*_*max*_ values of all immobilized trypsin biocatalysts were lower than that of free trypsin in solution. The amount of bound trypsin onto different magnetic support was calculated from Micro BCA assay (Table S1) and the final trypsin concentrations in the digestion buffer are listed in Table 1. To eliminate the influence of enzyme loading, *k*_*cat*_ is a better parameter to define and compare the reaction rate. It is the number of substrate molecules converted to product per enzyme molecule per second, and indicates how fast each enzyme molecule can catalyze the biotransformation. As shown in Table 1, the immobilized trypsin onto Fe_3_O_4_-NH_2_/PAA magnetic support exhibited comparable reaction turnover number to the benchmark and the highest *k*_*cat*_ value among in-house synthesized magnetic beads. The reaction rate of Fe_3_O_4_-NH_2_/PAA/trypsin was accelerated by 1.65-fold compared to free trypsin in solution, confirming the enhanced biocatalytic activity of trypsin after immobilization.

Among the in-house synthesized magnetic supports, immobilized trypsin onto Fe_3_O_4_-NH_2_/PAA exhibited superior activity. In this system, the positively charged Fe_3_O_4_-NH_2_ surface enabled uniform adsorption of PAA via electrostatic interaction, forming a thin and flexible spacer layer that subsequently allowed amide coupling with trypsin. This interfacial architecture likely stabilized enzyme active conformation and induced a favorable protein orientation that guides substrates toward the active site, resulting in an increased catalytic rate per enzyme molecule [47]. In contrast, Fe_3_O_4_-OH/PEI/PAA and Fe_3_O_4_-COOH/PEI/PAA likely form thicker multilayer coating, promoting multipoint attachment, excessive conformational constraints and diffusion resistance, which might be invoked as an explanation for depressed reaction rate. Therefore, Fe_3_O_4_-NH_2_/PAA was selected as the optimal magnetic support for subsequent application.

Moreover, the ratio *k*_*cat*_*/K*_*m*_ is a characteristic parameter to describe the catalytic efficiency. It was found that the *k*_*cat*_*/K*_*m*_ values of Fe_3_O_4_-NH_2_/PAA/trypsin were comparable to benchmark and both higher than that of free trypsin. It can be concluded that PAA as the spacer arm for trypsin immobilization onto Fe_3_O_4_ nanoparticles provides higher catalytic efficiency as a heterogeneous biocatalyst compared to free trypsin, while offering advantages such as magnetic separation and reusability as well, making it a promising platform for heterogeneous catalysis.

#### Protein digestion with free and immobilized trypsin

To further evaluate the catalytic performance of immobilized trypsin, the digestion efficiency of Fe_3_O_4_-NH_2_/PAA/trypsin and benchmark/trypsin was tested with the model protein cytochrome c and compared with that of free trypsin in solution. The generated peptide fragments at different digestion times were analyzed by HPLC-ESI-QTOF-MS/MS. The total ion chromatograms (TICs) of free and immobilized trypsin digestion of CYC are shown in Fig. S5. The exported peak lists were subjected to Mascot database searches to assess digestion quality based on sequence coverage and protein identification scores (probability-based significance) [48]. As shown in Fig. S5(d) and (e), all three enzymes achieved efficient digestion within the first 10–30 min, and CYC was completely digested at 1 h with free and immobilized enzyme. The free trypsin and Fe_3_O_4_-NH_2_/PAA/trypsin reached their maximum sequence coverage with high scores at 1 h. However, for free trypsin, both parameters declined with prolonged digestion times (≥ 4 h). In contrast, the immobilized trypsin maintained relatively stable sequence coverage after extended digestion. It can be concluded that Fe_3_O_4_-NH_2_/PAA/trypsin provides digestion efficiency comparable to free enzyme while offering improved stability and controllability, and is easy to magnetically separate, avoiding interference from residual enzymes during analysis.

Furthermore, the digestion performance for immobilized trypsin across different batches was evaluated to verify the batch-to-batch repeatability of the immobilization process. CYC was digested using three independently prepared batches of Fe_3_O_4_-NH_2_/PAA/trypsin and benchmark/trypsin at 37 °C for 10 min. Each batch digestion was performed in triplicate. As shown in Fig. S6, both immobilized trypsin exhibited good repeatability, with RSD ranging from 3.5% to 9.8% for both batch-to-batch and intra-batch replicates. The low experimental variation indicated excellent consistency of the immobilization process.

The reusability of immobilized trypsin was investigated using the same batch of immobilized trypsin for repeated CYC digestion under identical condition. After each reaction, the immobilized enzyme was magnetically separated, washed twice with 50 mM ammonium bicarbonate buffer (pH 8.0), and reused for the next cycle. It can be seen from Fig. S7 that the sequence coverage remained within a narrow fluctuation range for both Fe_3_O_4_-NH_2_/PAA/trypsin and benchmark/trypsin over five consecutive digestion cycles, indicating high operational stability and good reusability.

For storage stability, one batch of Fe_3_O_4_-NH_2_/PAA/trypsin, benchmark/trypsin, and free trypsin in solution, respectively, was prepared the same day and stored at 4 °C until use. Then samples were used for CYC digestion at different days (1, 7, 20, 30, and 60 days). Fig. S8 shows that the sequence coverage of Fe_3_O_4_-NH_2_/PAA/trypsin and benchmark/trypsin digestion batches remained relatively constant during 60 days of storage. However, the free trypsin showed a gradually decreasing trend over time, and the score decreased. These results revealed that storage stability of the immobilized trypsin was improved compared to that of free enzyme.

### Application II: Proteinase K-IMER for protein digestion

#### Kinetic parameters of immobilized and free proteinase K

After identifying Fe_3_O_4_-NH_2_/PAA as the optimal support for trypsin immobilization due to its excellent catalytic performance, the generality of this support was further evaluated by applying it to another model protease-Proteinase K. To this end, Proteinase K was immobilized onto Fe_3_O_4_-NH_2_/PAA and commercially available benchmark magnetic beads, and its catalytic behavior was compared with free Proteinase K in solution. Cytochrome c was employed as the substrate, and the residual undigested CYC was quantified by an LC-UV method. A dedicated 10-min gradient separation on Aeris Peptide C18 column enabled most digested peptides to elute near the void volume, thereby reducing interference and improving the accuracy of quantitation of undigested CYC (details of experimental conditions are given in Fig. S9). A calibration curve for CYC in the range of 0.05–2.5 mg mL^−1^ showed excellent linearity (R^2^ = 0.9992, Fig. S10).

Lineweaver-Burk plots were determined from triplicate digestion experiments (n = 3) to determine the kinetic parameters of free and immobilized proteinase K (Table 2, Fig. S11). Fe_3_O_4_-NH_2_/PAA/proteinase K exhibited the lowest *K*_*M*_ value, indicating enhanced substrate affinity and unhindered access to the active site, confirming that the Fe_3_O_4_-NH_2_/PAA magnetic support did not impose steric limitations. The *V*_*max*_ of Fe_3_O_4_-NH_2_/PAA/proteinase K was comparable to the benchmark and higher than free enzyme, demonstrating that immobilization did not compromise the catalytic capacity. Based on the actual immobilized enzyme amount obtained from the Micro BCA assay (Table S3), *k*_*cat*_ was calculated using Eq. (2). Notably, Fe_3_O_4_-NH_2_/PAA/proteinase K showed a 1.82-fold increase in *k*_*cat*_ compared to free enzyme, indicating a higher catalytic turnover per enzyme molecule. This suggests that a lower amount of immobilized enzyme is sufficient to achieve the same reaction rate as free enzyme, enhancing the cost-efficiency of the biocatalyst. In terms of catalytic efficiency (*k*_*cat*_*/K*_*m*_), Fe_3_O_4_-NH_2_/PAA/proteinase K showed the highest value, further confirming the enhanced catalytic efficiency of proteinase K after immobilization.

**Table 2:** Comparison of kinetic parameters for different proteinase K-IMER and free proteinase K

Parameter	Fe_3_O_4_-NH_2_/PAA/proteinase K	Benchmark/proteinase K	Free proteinase K
*K*_*m*_ [M]	(1.55 ± 0.05) × 10^–5^	(6.34 ± 0.67) × 10^–5^	(1.97 ± 0.37) × 10^–5^
*V*_*max*_ [M·s^−1^]	(4.41 ± 0.14) × 10^–8^	(1.48 ± 0.15) × 10^–7^	(2.96 ± 0.51) × 10^–8^
*K*_*cat*_ [s^−1^]	(4.82 ± 0.26) × 10^–2^	(1.26 ± 0.15) × 10^–1^	(1.71 ± 0.30) × 10^–2^
*K*_*cat*_*/K*_*m*_ [M^−1^·s^−1^]	(3.10 ± 0.20) × 10^3^	(1.98 ± 0.32) × 10^3^	(8.70 ± 2.22) × 10^2^
Final proteinase K concentration [M]	(9.14 ± 0.41) × 10^–7^	(1.17 ± 0.07) × 10^–6^	1.73 × 10^–6^

#### Immobilized proteinase K for non-specific protein digestion

**Fig. 5:**
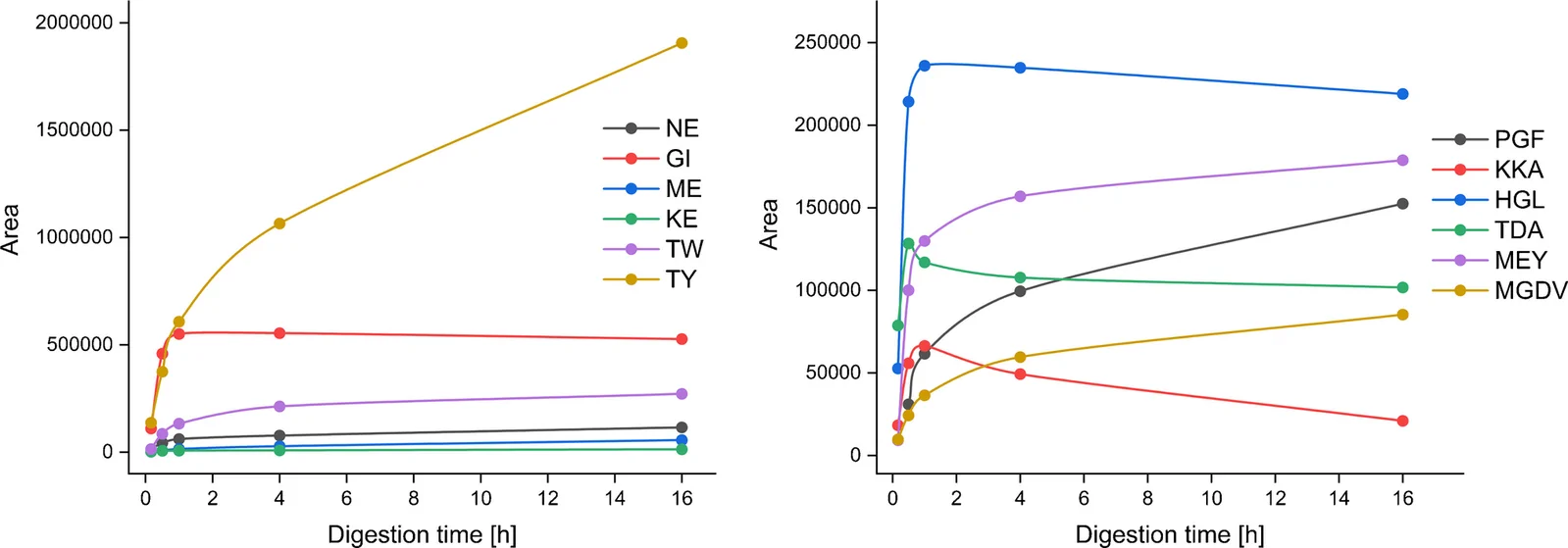
Kinetic curve of detected small peptides from CYC digestion by Fe_3_O_4_-NH_2_/PAA/proteinase K

Next to the activity test with chromatographic characterization, proteinase K-immobilized magnetic beads were also tested for non-specific proteolysis using CYC. Unlike trypsin-based proteomics aiming for specific peptide mapping, proteinase K digestion is highly unspecific and generates a variety of short peptides that are valuable for endogenous peptidome profiling and bioactive peptide discovery [36, 49]. Theoretical cleavage sites of proteinase K were first predicted using the Expasy PeptideCutter and PeptideMass tools, and the corresponding predicted short peptides were subsequently searched against the experimental LC-MS/MS data. Table S5 and Figs. S12 and S13 show an overview of the detected small-peptide precursors (mainly di- and tripeptides) generated by Fe_3_O_4_-NH_2_/PAA/proteinase K, overlays of extracted ion chromatograms (XICs) of small peptides obtained from CYC digestion at different digestion time and exemplary MS/MS spectrum of a model dipeptide. The digestion kinetic curves of identified small peptides from CYC digestion with different digestion time were plotted (Fig. 5). The relative abundances of most small peptides increased with time, reaching a maximum after 4 h of digestion. A few small peptides peaked around 1 h and then decreased with prolonged digestion. These results demonstrate the feasibility of using immobilized proteinase K for controlled non-specific digestion. Although only a limited number of fragments were identified here, more dipeptides and tripeptides can be expected when digesting diverse protein substrates, which could provide a valuable resource for short-peptide identification and peptidomics analysis.

The storage stability of Fe_3_O_4_-NH_2_/PAA/proteinase K was evaluated based on its retained digestion capability toward the CYC substrate. As shown in Fig. S14, the relative digestion activity was calculated from the normalized summed peak areas of identified peptides after 1 h digestion by immobilized proteinase K. The results indicate the good storage stability of the immobilized proteinase K.

### Application III: RNase T1-IMER for RNA digestion

#### Digestion kinetics of RNA using immobilized RNase T1

**Fig. 6:**
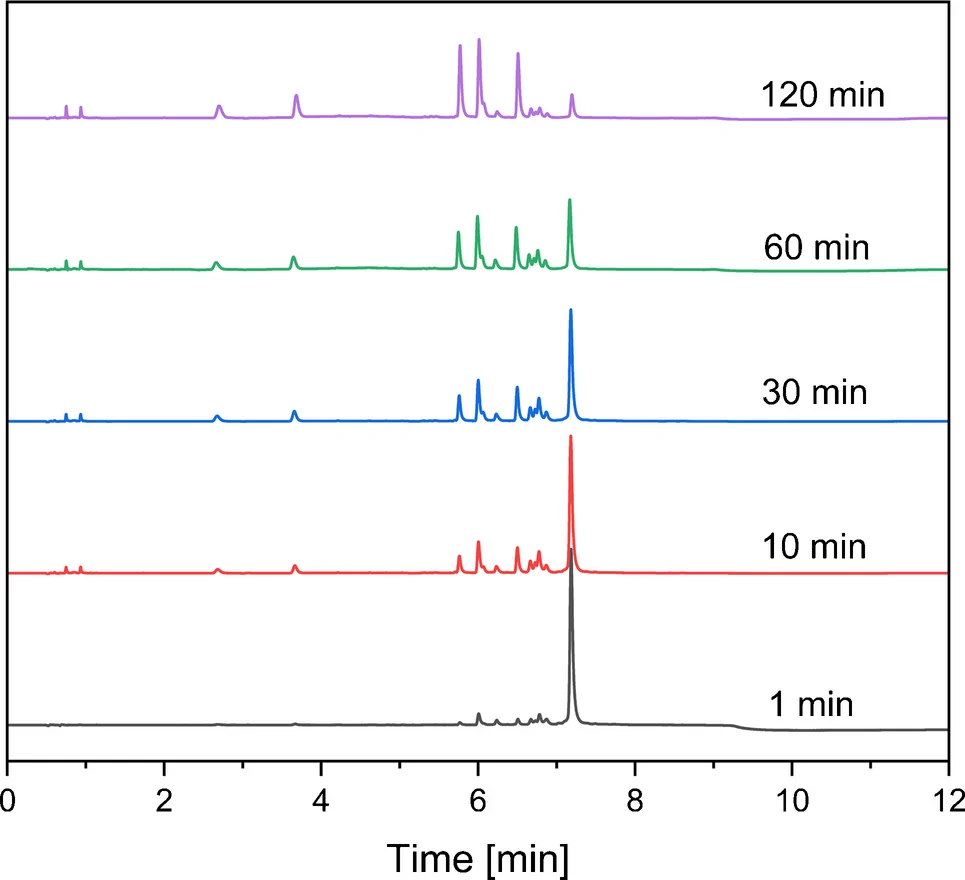
HPLC-UV chromatogram of RNA digestion by Fe_3_O_4_-NH_2_/PAA/RNase T1 after different digestion times

The applicability of Fe_3_O_4_-NH_2_/PAA magnetic support as IMER was further explored by extending it to a ribonuclease-RNase T1. A 15-mer RNA standard (AUCGGCCAUGCGAAU) was digested using immobilized RNase T1, and the digestion kinetics of RNA was evaluated on LC-UV analysis with different digestion time. The separation was performed with a YMC Triart C18 column (100 × 2 mm, 1.9 μm). Mobile phase A consisted of H_2_O with 50 mM triethylammonium acetate at pH 7 and mobile phase B consisted of 75/25 (v/v) ACN/water with 50 mM triethylammonium acetate at pH 7 (0–2 min,10% B; 2–8 min, 10–65% B; 8–8.1 min, 65–10% B; 8.1–12 min, 10% B). The flow rate was 0.4 mL min^−1^ and detection was performed at 260 nm. Figure 6 shows the time-dependent LC-UV chromatograms of RNA digestion profile (1–120 min) catalyzed by immobilized RNase T1. Distinct chromatographic changes were observed as the reaction progressed, confirming the bioactivity of immobilized RNase T1 and controllable digestion by magnetic separation. After 120 min of digestion, the peak area of the intact RNA substrate decreased by 83% (Fig. S15), demonstrating the high digestion efficiency of Fe_3_O_4_-NH_2_/PAA/RNase T1.

#### Identification of RNA digests by HILIC-ESI-QTOF-MS

**Fig. 7:**
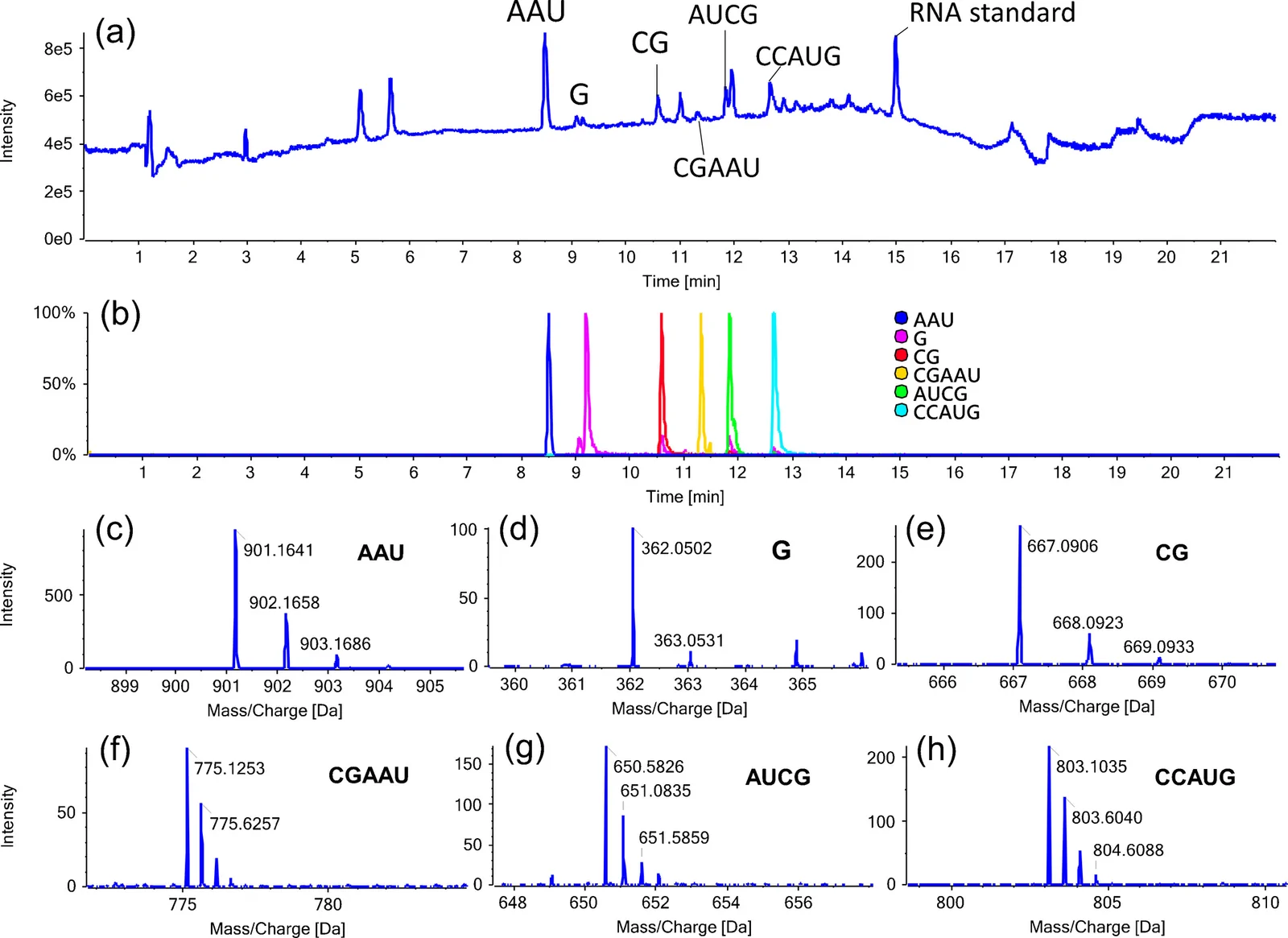
TIC (**a**) and XIC (**b**) of RNA digestion by Fe_3_O_4_-NH_2_/PAA/RNase T1 at digestion time of 1 h; TOF-MS spectra (**c**–**h**) of digestion fragments of RNA standard (AUCGGCCAUGCGAAU)

For further characterization of the digestion products, the reaction mixture collected after 1 h of incubation was analyzed by HILIC-ESI-QTOF-MS. RNase T1 specifically cleaves single-stranded RNA after guanosine (G) residues [6]. The enzyme catalyzes a nucleophilic attack by the 2′-OH of the ribose, forming a 2′,3′-cyclic phosphate intermediate, which is subsequently hydrolyzed to yield oligoribonucleotides with 3′ phosphate and 5′ hydroxyl terminus. The resulting RNA fragments were separated by HILIC and analyzed by high-resolution TOF mass spectrometry. The extracted-ion chromatograms and mass spectra of the digestion fragments are shown in Fig. 7. For the identification of RNA (Fig. S16) and RNA digestion fragments, the monoisotopic mass of the identified components in the experiment was compared to the theoretical oligoribonucleotide components. The identification results are summarized in Table S6 and all theoretical RNase T1 digestion fragments were detected in the experimental data, demonstrating that RNase T1 retained its sequence-specific catalytic activity after immobilization. Overall, the LC-MS results document the new immobilized RNase T1 on magnetic support has adequate bioactivity for RNA digestion while offering facile separation and potential reusability.

The storage stability of Fe_3_O_4_-NH_2_/PAA/RNase T1 was evaluated based on its retained digestion capability toward the RNA substrate. As shown in Fig. S17, the immobilized RNase T1 maintained high relative activity after storage at 4 °C for 30 days, demonstrating the good storage stability of Fe_3_O_4_-NH_2_/PAA/RNase T1.

#### Applicability to mRNA digestion

**Fig. 8:**
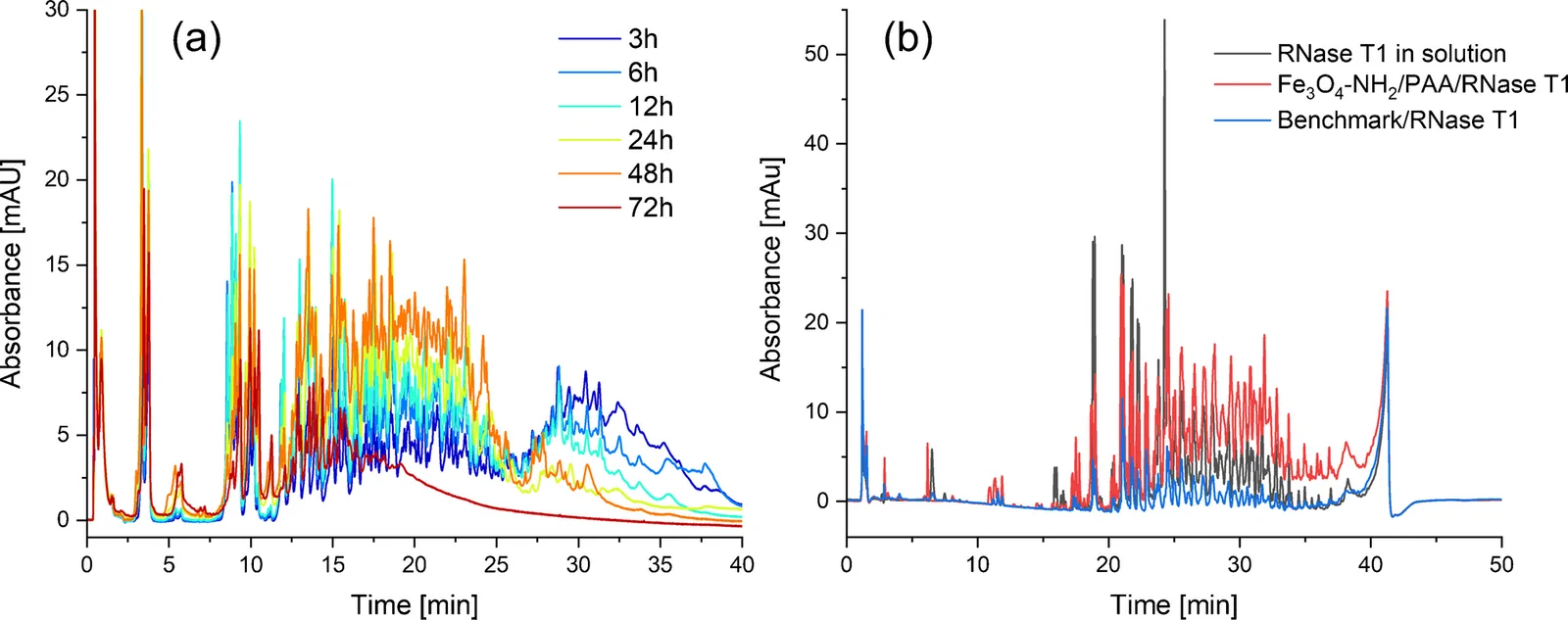
HILIC-UV profiles of mRNA digestion by Fe_3_O_4_-NH_2_/PAA/RNase T1 IMER after different digestion times (**a**), comparison of HILIC-UV profiles of mRNA digests generated by free RNase T1 in solution, Fe_3_O_4_-NH_2_/PAA/RNase T1 IMER and benchmark/RNase T1 IMER at 16 h (**b**)

In addition to characterization of the immobilized RNase T1 using a small RNA standard, digestion of a real sample, an mRNA vaccine, was performed to evaluate its applicability for the intended use of the developed immobilized RNase T1. mRNA was first extracted from mRNA-LNP formulation and then digested by Fe_3_O_4_-NH_2_/PAA/RNase T1 at different digestion times. Digestion products were separated by an Acquity UPLC BEH Amide (50 × 2.1 mm, 1.7 μm) column. The mobile phase consisted of H_2_O with 10 mM ammonium acetate in channel A and 90/10 (v/v) acetonitrile/water with 10 mM ammonium acetate in channel B (unadjusted pH). The gradient profile was as follows: 0–40 min, 5% A; 40–40.1 min, 5–60% A; 40.1–60 min, 5% A. The flow rate was 0.3 mL min^−1^, injection volume was 5 µL and detection was performed at 260 nm. Figure 8a shows the time-dependent HILIC-UV profiles obtained for Fe_3_O_4_-NH_2_/PAA/RNase T1-digested mRNA. It can be seen that the number of mRNA digestion fragments increases with longer digestion times. As the digestion progresses, the peaks shift to shorter retention times, suggesting that the mRNA is broken down into smaller fragments, which interact less strongly with the stationary phase and elute earlier. Between 24 and 48 h of digestion, the peak shape showed only slight differences, indicating that digestion was essentially complete after about 24 h. The differences between the 48- and 72-h digestions could be due to non-enzymatic degradation at 37 °C. Overall, the chromatogram reflects the expected increase in fragmentation with longer digestion times, confirming the applicability of Fe_3_O_4_-NH_2_/PAA/RNase T1 for mRNA digestion.

In addition, mRNA digestion was carried out using free RNase T1 and different RNase T1 IMERs for 16 h to further evaluate the catalytic performance of immobilized RNase T1. The digests were separated using a Waters Acquity UPLC BEH Amide column (150 × 2.1 mm, 1.7 μm) with a 50-min gradient. Figure 8b indicates that Fe_3_O_4_-NH_2_/PAA/RNase T1 exhibits higher digestion efficiency than benchmark/RNase T1 and a digestion efficiency comparable to that of free RNase T1. Moreover, it enables controllable digestion via magnetic separation, thereby preventing interference from residual enzyme during subsequent analysis.

## Conclusion

Fe_3_O_4_ nanoparticles bearing different functional groups modified with polyelectrolyte layers were synthesized as magnetic supports for enzyme immobilization. The magnetic platform materials were characterized by HIM, FT-IR, zeta potential, and EDX. Firstly, trypsin as the model enzyme was immobilized onto different magnetic supports, the bioactivity of immobilized trypsin and the loading amount of trypsin onto different magnetic beads were compared and evaluated systematically. The optimal IMER—Fe_3_O_4_-NH_2_/PAA/trypsin was elected due to its excellent surface bioreactivity and loading capacity. PAA as the monolayer provides higher catalytic efficiency as a heterogeneous biocatalyst compared to free trypsin, and also for enzyme immobilization via amide coupling. The immobilized trypsin also demonstrated good reproducibility and stability. Subsequently, the application of the Fe_3_O_4_-NH_2_/PAA immobilization platform was extended to non-specific proteases-proteinase K. Based on the predicted cleavage sites obtained from theoretical digestion tools, many small peptides were experimentally detected, which indicates that immobilized non-specific enzymes can be used to generate small peptide mixtures, offering reference value for peptidomics profiling. Furthermore, a 15mer-RNA standard was digested by immobilized RNase T1, and the reaction could be easily terminated within seconds by magnetic separation, eliminating enzyme interference. All theoretical RNase T1 digestion fragments were confirmed by HILIC-ESI-QTOF-MS/MS analysis, indicating that the enzyme retained its sequence-specific catalytic activity after immobilization. In addition, the mRNA digestion results demonstrated the applicability of developed immobilized RNase T1 in a real sample. Overall, these results confirm the broad applicability and versatility of Fe_3_O_4_-NH_2_/PAA magnetic support in enzyme immobilization and biopharmaceutical applications, offering controllable biocatalytic digestion and facile magnetic separation.

## Supplementary Information

Below is the link to the electronic supplementary material.

Supplementary file1 (DOCX 14.2 MB)

## Data Availability

The authors confirm that the data supporting the findings of this study are available within the article or its supplementary materials.
