# Supplementary material for: Trypsin-, proteinase K-, and ribonuclease T1-functionalized magnetic nanoparticle-immobilized enzyme reactors based on polyelectrolyte multilayer platform for biopharmaceutical analysis
Source: Anal Bioanal Chem. 2026 May 29;418(23):7873–87. doi: 10.1007/s00216-026-06580-5 (PMC13744482; doi:10.1007/s00216-026-06580-5)
Supplement: Supplementary file 1 [file 216_2026_6580_MOESM1_ESM.docx]

**Supplementary Material**

**Trypsin-, Proteinase K-, and Ribonuclease T1-Functionalized** **Magnetic nanoparticle-immobilized enzyme reactors based on polyelectrolyte multilayer platform for biopharmaceutical analysis**

Yachao Hao^a^, Niklas Carstensen^a^, Ronny Loeffler^b^, Benjamin Junker-Reiss^b,c^, Dominique Lunter^a^, Michael Lämmerhofer^a#^

^a^ Institute of Pharmaceutical Sciences, Pharmaceutical (Bio-)Analysis, University of Tübingen, Auf der Morgenstelle 8, 72076 Tübingen, Germany

^b^ Center for Light-Matter Interaction, Sensors & Analytics (LISA^+^), University of Tübingen, Auf der Morgenstelle 15, D-72076 Tübingen, Germany

^c^ Institute of Physical and Theoretical Chemistry, University of Tübingen, Auf der Morgenstelle 15, D-72076 Tübingen, Germany

\# Author for correspondence:

E-mail: [michael.laemmerhofer@uni-tuebingen.de](mailto:michael.laemmerhofer@uni-tuebingen.de)

**Fig. S1** FT-IR images of Fe_3_O_4_-OH (a), Fe_3_O_4_-OH/PEI (b), Fe_3_O_4_-OH/PEI/PAA (c), Fe_3_O_4_-COOH (d), Fe_3_O_4_-COOH/PEI (e) and Fe_3_O_4_-COOH/PEI/PAA (f)

**Fig. S2** Zeta potential of different magnetic supports after each step of synthesis and surface modification

**Fig. S3** EDX spectrum of Fe_3_O_4_-NH_2_/PAA/Trypsin (a), Fe_3_O_4_-NH_2_/PAA/Proteinase K (b) and Fe_3_O_4_-NH_2_/PAA/RNase T1 (c)

**The determination of the amount of immobilized protein by Micro BCA assay**

The amount of immobilized protein on magnetic beads was determined directly with Micro BCA Assay. Due to the advantage of low protein to protein response variability of the Micro BCA kit, the BSA standard provided in the kit was used as the assay standard for quantitative comparison of the immobilized protein loading across different magnetic beads. The protein quantity was determined by absorbance measurement at 562 nm against the standard curve. To correct for potential interference from magnetic beads and avoid false positive signal absorption, magnetic beads without immobilization (processed in parallel under identical assay conditions) were used as controls, and the corresponding absorbance background was subtracted from all measurements. Thus, a calibration function ranging from 0.5 to 20 μg mL^-1^ was established in triplicate (n=3), with satisfactory correlation coefficients (R^2^ = 0.9938, **Fig. S4**).

The results of the amount of immobilized trypsin on different magnetic beads were shown in **Table S1**. Among all supports evaluated, Fe_3_O_4_-NH₂/PAA/Trypsin exhibited the highest immobilized trypsin content (52.7 ± 2.7 μg mg^-1^ beads), indicating superior surface reactivity and anchoring capacity. In contrast, Fe_3_O_4_-OH/PEI/PAA displayed the lowest loading (41.3 ± 1.8 μg mg^-1^ beads). It could be due to the low density of reactive hydroxyl groups on the as-synthesized Fe_3_O_4_ surface. The insufficient number of anchoring sites restricted the modification of the polymer layer, thereby limiting the subsequent conjugation of trypsin.

In addition, to elucidate the possible adsorption mechanism of Fe_3_O_4_-NH₂/PAA toward the enzyme, physical adsorption of trypsin without EDC/NHS activation as control experiments were performed under identical immobilization conditions **(Table S2)**. The results showed that EDC/NHS-mediated covalent coupling significantly enhanced protein immobilization on both Fe_3_O_4_-NH₂/PAA and Benchmarking supports. For the benchmarking support, the amount of immobilized trypsin obtained via covalent coupling (43.3 ± 1.2 μg mg^-1^) was substantially higher than that from physical adsorption (9.7 ± 0.5 μg mg^-1^), indicating that covalent attachment is the favourable immobilization mechanism in this system. In contrast, the Fe_3_O_4_-NH₂/PAA support still retained a relatively high amount of trypsin through physical adsorption (35.1 ± 2.6 μg mg^-1^). It is reasonable that electrostatic interactions and [hydrogen bonding](https://www.sciencedirect.com/topics/materials-science/hydrogen-bonding) were favourable for adsorption due to the presence of abundant surface carboxyl groups from PAA layer which can provide abundant [hydrogen bonding](https://www.sciencedirect.com/topics/chemistry/hydrogen-bonding) sites and facilitate protein adsorption. In addition, coordination interactions between enzyme and Fe sites may also contribute to the adsorption process. The above results suggest that trypsin immobilization onto the Fe_3_O_4_-NH₂/PAA support can be mainly ascribed to covalent coupling, hydrogen bonding, electrostatic and coordination interactions. The covalent coupling assures that there is no significant leakage of the enzyme during application.

**Fig. S4** Micro BCA assay: Calibration function of BSA standard

**Table S1.** Comparison of the amount of immobilized trypsin

| Trypsin-IMER | Mean mass of trypsin per magnetic beads [μg mg^-1^] | Concentration of immobilized trypsin [mol L^-1^] |
| --- | --- | --- |
| Fe_3_O_4_-OH/PEI/PAA/Trypsin | 41.3 ± 1.8 | (4.7 ± 0.2) ×10^-6^ |
| Fe_3_O_4_-COOH/PEI/PAA/Trypsin | 44.7 ± 1.7 | (4.3 ± 0.2) ×10^-6^ |
| Fe_3_O_4_-NH_2_/PAA/Trypsin | 52.7 ± 2.7 | (5.6 ± 0.3) ×10^-6^ |
| Benchmarking/Trypsin | 43.3 ± 1.2 | (4.5 ± 0.2) ×10^-6^ |

**Table S2.** Comparison of trypsin immobilization via covalent coupling and physical adsorption

| Immobilization method | Mean mass of trypsin on Fe_3_O_4_-NH_2_/PAA/Trypsin [μg mg^-1^] | Mean mass of trypsin on Benchmarking/Trypsin [μg mg^-1^] |
| --- | --- | --- |
| EDC/NHS covalent coupling | 52.7 ± 2.7 | 43.3 ± 1.2 |
| Physical adsorption | 35.1 ± 2.6 | 9.7 ± 0.5 |

**Table S3.** Comparison of the amount of immobilized proteinase K

| Proteinase K-IMER | Mean mass of proteinase K per magnetic beads [μg mg^-1^] | Concentration of immobilized proteinase K [mol L^-1^] |
| --- | --- | --- |
| Fe_3_O_4_-NH_2_/PAA/Proteinase K | 42.2 ± 1.9 | (3.7 ± 0.2) ×10^-6^ |
| Benchmarking/Proteinase K | 54.3 ± 3.4 | (4.7 ± 0.3) ×10^-6^ |

**Table S4.** Comparison of the amount of immobilized RNase T1

| RNase T1-IMER | Mean mass of RNase T1 per magnetic beads [μg mg^-1^] | Concentration of immobilized RNase T1 [mol L^-1^] |
| --- | --- | --- |
| Fe_3_O_4_-NH_2_/PAA/RNase T1 | 16.6 ± 0.6 | (3.8 ± 0.1) ×10^-6^ |
| Benchmarking/RNase T1 | 15.2 ± 0.9 | (3.5 ± 0.2) ×10^-6^ |

**Protein digestion with free and immobilized trypsin**

**Fig. S5** The total ion chromatograms (TICs) of CYC digested by free trypsin in solution (a), Fe_3_O_4_-NH_2_/PAA/Trypsin (b) and Benchmark/Trypsin (c) at different digestion time, along with the corresponding sequence coverage (d) and score (e) obtained under each condition

**Repeatability and stability of immobilized trypsin**

**Fig. S6** Batch-to-batch repeatability of immobilized trypsin

**Fig. S7** Reusability of immobilized trypsin

**Fig. S8** Storage stability of Fe_3_O_4_-NH_2_/PAA/Trypsin (a), Benchmark/Trypsin (b) and free trypsin in solution (c)

**Chromatographic characterization of proteinase K activity**

**Fig. S9** Chromatogram of HPLC-UV determination of undigested CYC digestion for proteinase K kinetic study (Mobile phase A: 0.1 %(v/v) TFA in H_2_O; mobile phase B: 0.1 %(v/v) TFA in acetonitrile; flow rate: 1 mL min^-1^; column temperature: 40 °C)

**Fig. S10** Calibration function for CYC analysed in the course of the enzyme kinetics study

**Fig. S11** Lineweaver-Burk diagrams of different proteinase K-IMER and free proteinase K

**Digestion kinetics of small peptides of CYC digestion by Fe_3_O_4_-NH_2_/PAA/Proteinase K**

**Table S5.** Summary of detected small peptides from CYC digestion by Fe_3_O_4_-NH_2_/PAA/Proteinase K after 4 h of digestion time (*m/z* refers to the monoisotopic mass)

| Resulting small peptide sequence | Ion type | Calculated *m/z* | Measured *m/z* | Error [ppm] | RT [min] |
| --- | --- | --- | --- | --- | --- |
| NE | [M+H]⁺ | 262.1034 | 262.1031 | -1.11 | 0.80 |
| GI | [M+H]⁺ | 189.1234 | 189.1233 | -0.53 | 1.94 |
| ME | [M+H]⁺ | 279.1009 | 279.1010 | 0.29 | 1.04 |
| KE | [M+H]⁺ | 276.1554 | 276.1557 | 0.98 | 0.73 |
| TW | [M+H]⁺ | 306.1448 | 306.1449 | 0.10 | 5.09 |
| TY | [M+H]⁺ | 283.1289 | 283.1288 | -0.25 | 1.38 |
| PGF | [M+H]⁺ | 320.1605 | 320.1605 | -0.09 | 4.73 |
| KKA | [M+H]⁺ | 346.2449 | 346.2448 | -0.23 | 0.76 |
| HGL | [M+H]⁺ | 326.1823 | 326.1822 | -0.21 | 3.96 |
| TDA | [M+H]⁺ | 306.1296 | 306.1295 | -0.33 | 0.84 |
| MEY | [M+H]⁺ | 442.1643 | 442.1639 | -0.77 | 4.16 |
| TNE | [M+H]⁺ | 363.1510 | 363.1512 | 0.41 | 0.82 |
| MGDV | [M+H]⁺ | 421.1752 | 421.1754 | 0.64 | 0.81 |

Expasy Bioinformatics Resource Portal predicts potential substrate cleavage sites for Proteinase K in a given protein sequence based on PeptideCutter (<https://web.expasy.org/peptide_cutter/>) and PeptideMass (<https://web.expasy.org/peptide_mass/>) tool.

**Fig. S12** UHPLC–ESI-QTOF-MS/MS extracted ion chromatograms (XICs) of small peptides obtained from CYC digestion with Fe_3_O_4_-NH_2_/PAA/proteinase K at digestion times of 10 min, 30 min, 1 h, 4 h and 16 h

**Fig. S13** Exemplary MS spectrum (a) and MS/MS spectrum of dipeptide GI. Determined b-/y-ions with PeakView (c) after CYC digestion by Fe_3_O_4_-NH_2_/PAA/Proteinase K for 4 h

**Fig. S14** Storage stability of Fe_3_O_4_-NH_2_/PAA/Proteinase K

**Digestion kinetics of RNA using immobilized RNase T1**

**Fig. S15** Digestion efficiency of RNA catalyzed by Fe_3_O_4_-NH_2_/PAA/RNase T1

**Identification of RNA digests by HILIC-ESI-QTOF-MS**

**Fig. S16** TIC (a) and TOF-MS spectrum (b) of RNA standard (AUCGGCCAUGCGAAU); (c, d): Magnified ESI-MS spectra in negative mode of the most abundant signals from the corresponding [M-nH]^n-^ peak group

**Table S6.** Summary of identification results of RNA digestion fragments

| Sequence | Ion type | Calculated m/z | Measured m/z | Error [ppm] | RT [min] |
| --- | --- | --- | --- | --- | --- |
| AAU | [M-H]^-^ | 901.1657 | 901.1641 | -1.78 | 8.50 |
| G | [M-H]^-^ | 362.0497 | 362.0502 | 1.38 | 9.20 |
| CG | [M-H]^-^ | 667.0907 | 667.0906 | -0.15 | 10.59 |
| CGAAU | [M-2H]^2-^ | 775.1235 | 775.1253 | 2.32 | 11.35 |
| AUCG | [M-2H]^2-^ | 650.5813 | 650.5826 | 2.00 | 11.85 |
| CCAUG | [M-2H]^2-^ | 803.1007 | 803.1035 | 3.49 | 12.67 |
|  |  |  |  |  |  |
| AUCGGCCAUGCGAAU | [M-4H]^4-^ | 1192.1633 | 1192.1686 | 4.45 | 14.99 |
|  | [M-3H]^3-^ | 1589.8867 | 1589.8921 | 3.40 |  |

**Fig. S17** Storage stability of Fe_3_O_4_-NH_2_/PAA/RNase T1
